# Characterizing Epidemiological Trends and Associated Factors of Japanese Encephalitis in China: Insights From a 17–Year National Surveillance Analysis

**DOI:** 10.1155/tbed/8854015

**Published:** 2025-12-28

**Authors:** Junze Du, Rui Li, Peng Li, Ting Fu, Zurong Yang, Bo Wen, Zhijia Sun, Guzhengyue Zheng, Haiyan Zhou, Hongxia Tan, Kun Liu

**Affiliations:** ^1^ Department of Epidemiology, School of Public Health, The Ministry of Education Key Lab of Hazard Assessment and Control in Special Operational Environment, The Shaanxi Provincial Key Laboratory of Environmental Health Hazard Assessment and Protection, The Fourth Military Medical University, Xi’an, 710032, China, fmmu.edu.cn; ^2^ Centre for Disease Prevent and Control in Northern Theater Command, 6 Long Shan Road, Shenyang, 110034, China; ^3^ Lintong Rehabilitation and Convalescent Center, Xi’an, 710600, China; ^4^ Department of Radiation Oncology, Air Force Characteristic Medical Center, Air Force Medical University, Beijing, China, fmmu.edu.cn; ^5^ School of Medicine, Xizang Minzu University, No. 6 Wenhui East Road Weicheng District, Xianyang, 712082, Shaanxi, China, xzmu.edu.cn

**Keywords:** Bayesian spatiotemporal hierarchy model, Japanese encephalitis, national surveillance, risk factors, spatial epidemiology

## Abstract

Japanese encephalitis (JE) is a mosquito‐borne infectious disease that is primarily endemic in Asia and the Western Pacific region. In recent years, the epidemiological profile of JE in China has undergone significant transformations, posing novel challenges to disease control and prevention. To systematically evaluate these changes, we analyzed nationwide longitudinal surveillance data on JE incidence from 2004 to 2020. Bayesian spatiotemporal hierarchy model and age–period–cohort analysis were employed to explore long‐term trends and transmission dynamics, and the geographical detector model was used to assess the synergistic effects of meteorological, ecological, and socioeconomic factors on the distribution of JE. During the study period, a total of 43,857 JE cases were reported in mainland China, with an incidence rate per 100,000 population declining from 0.42 to 0.02. Spatially, traditional hyperendemic areas remained concentrated in southwest (Sichuan, Guizhou, Yunnan, and Chongqing), while significant northward expansion was observed in the northwestern China of Gansu and Shaanxi provinces. Our models indicated that spatiotemporal heterogeneity in JE transmission was strongly influenced by the interaction effects between socioeconomic development and meteorological variables, particularly the medical level, GDP per capita, and education level. These findings underscore the need for spatially adaptive and age‐specific public health strategies in response to changing JE risks under socioeconomic and environmental transitions.

## 1. Background

Japanese encephalitis (JE) is a mosquito‐borne viral meningoencephalitis caused by JE virus (JEV), which belongs to the family *Flaviviridae* and genus *Flavivirus* [1]. The disease is mainly transmitted by *Culex* mosquitoes (primarily *Culex tritaeniorrhynchus* and *Culex vishnui*) among birds and pigs, which serve as amplifying hosts, whereas humans act as dead‐end hosts [2]. JE was first documented in Japan in 1871 and later spread throughout Asia and Oceania such as India, China, Korea, Indonesia [3, 4], and Australia [5]. According to a mathematical modeling study, JEV infections were estimated to have caused 100,308 JE cases and 25,125 deaths globally in 2015 [6].

JE has imposed a substantial disease burden on China. Between 1963 and 1975, China experienced major JE epidemics, with an average annual incidence ranging from 10 to 15 cases per 100,000 population. The incidence reached a peak of 20.92 per 100,000 population in 1971 [7]. After effective interventions and immunization strategies, the incidence of JE has experienced a considerable decrease in mainland China. Nevertheless, despite the implementation of a national JE Expanded Program on Immunization (EPI) in 2008, outbreaks in western China during 2017 and 2018 underscore that JE remains a persistent and significant public health concern [8, 9]. JE predominantly affects children under 15 years of age, with a casefatality rate of 20%–30% and permanent neurological, cognitive, or behavioral sequelae occurring in ~30%–50% of survivors [6–11]. Given the absence of specific treatment for JE, prevention and control of JE remain the most effective strategies for reducing disease burden [12, 13].

As a mosquito‐borne disease, JE has significant associations with meteorological, ecological, and socioeconomic factors and exhibits marked spatial heterogeneity in transmission patterns [14, 15]. Previous studies across different provinces in China have examined the factors influencing JE, revealing regional disparities in their contributing influences [16–19]. However, research on the overall spatiotemporal distribution patterns of JE across mainland China and its underlying drivers remains limited. The spatiotemporal dynamics of socioeconomic, meteorological, and ecological factors influencing JE transmission have not been comprehensively elucidated, implying a critical gap in understanding the complex interplay between environmental and anthropogenic drivers in disease epidemiology.

In this study, we utilized nationwide longitudinal surveillance data on JE incidence across China from 2004 to 2020 to systematically analyze trends in JE transmission, and a multivariate modeling framework was applied to quantify the synergistic effects of meteorological, ecological, and socioeconomic factors on the spatiotemporal distribution of JE. Our findings identify key drivers underlying the spatiotemporal heterogeneity of JE transmission risk across China and offer evidence‐based insights for formulating targeted public health interventions.

## 2. Materials and Methods

### 2.1. Study Design and Data Source

We conducted a retrospective study to assess the evolving spatiotemporal epidemiological patterns of JE in China and to identify the factors influencing its distribution. In this study, the reported absolute cases and rates per 100,000 population of JE were obtained from the Public Health Science Data Repository of China (https://www.phsciencedata.cn/Share/index.jsp), covering the period from 2004 to 2020. Following the outbreak of severe acute respiratory syndrome (SARS) in 2003, the Chinese government established the Notifiable Infectious Diseases Reporting Information System (NIDRIS) to conduct real‐time surveillance of the dynamic trends in infectious diseases. Because the available data ended in 2020, we included data from 2004 to 2020 for analysis. The analyzed geographical units include 31 provincial administrative divisions in mainland China (excluding Hong Kong, Macau, and Taiwan due to inaccessible data), comprising 22 provinces, 5 autonomous regions, and 4 municipalities (hereafter referred to as 31 provinces), and these provinces were grouped into northern China (16 provinces) and southern China (15 provinces) [20] (Table S1). The JE cases in the study were diagnosed by healthcare professionals in accordance with the Chinese National Health Commission guidelines (WS214–2008). A suspected JE case was defined as an individual with an acute onset of fever, headache, and projectile vomiting, which could progress to impaired consciousness. This clinical presentation needed to be accompanied by a compatible epidemiological history (residence in or travel to a JE‐endemic area during the mosquito season) and leukocytosis. A laboratory‐confirmed case was defined as a suspected case that also met at least one of the following criteria: detection of anti‐JEV IgM antibody in serum or cerebrospinal fluid (note: this criterion applies only to unvaccinated individuals); seroconversion or a ≥ 4‐fold increase in JEV‐specific IgG or neutralizing antibody titers between acute and convalescent sera; isolation of JEV; or detection of JEV‐specific nucleic acid. All cases were systematically recorded in the NIDRIS.

Based on the existing literature and public health significance of environmental determinants related to JE, the meteorological, ecological and socioeconomic factors were included in this study (Table S2). Specifically, meteorological data (temperature, relative humidity, precipitation, and wind speed) were sourced from the China Meteorological Data Sharing Service Center (http://data.cma.cn/). Annual averages of the meteorological variables were calculated using ArcGIS 10.8 (ESRI Inc, Redlands, CA). Ecological data were obtained from the 30 m Annual Land Cover Dataset of China [21], and the annual averages of socioeconomic data (per capita GDP, population density, pig density, built‐up area, education level, medical level, and RPT) for 31 provinces during 2004–2020 were obtained from the China Statistical Yearbook (http://www.stats.gov.cn/). These indicators were aggregated at the provincial level.

### 2.2. Statistical Analysis

#### 2.2.1. Bayesian Space–Time Hierarchy Model (BSTHM)

BSTHM was used to identify the spatiotemporal characteristics of JE incidence in 31 provinces across mainland China from 2004 to 2020. Poisson distribution was used to evaluate the count of cases *y*_*it*_ and the potential risk population *n*_*i*_ as *y*_*it*_ ∼ *Poisson* (*n*_*it*_ *u*_*it*_). The underlying risk *u*_*it*_ is modeled as follows:

loguit=α+si+b0t∗+νt+b1it∗+εit,

where *u*_*it*_ is the spatial relative risk (RR) of each province *i* (*i* = 1, 2, …, 31) in year *t* (*t* = 1, 2, …, 17), and *α* is used to indicate the spatial disease risks in mainland China during 2004–2020. The spatial term *s*_*i*_ represents the spatial heterogeneity of the disease risks during the selected period. Similarly, (*b*_0_ *t* ^⁎^ + *v*_*t*_) stands for the overall temporal trend, while *t* ^⁎^ denotes the time point at the midpoint of the observation period, and *b*_*1i*_ reveals the deviation from *b*_0_, representing the local temporal trend. Finally, the term *ε*_*it*_ is a Gaussian random noise term representing random time effect.

To assess the spatial heterogeneity of RR for JE, the specific two‐phase criteria were applied; local temporal trends were investigated utilizing the model’s posterior estimation parameters. Specifically, during the initial phase, provinces were classified as follows: a hot spot if the posterior probability [exp(s_i_) > 1 | data] ≥ 0.8, a cold spot if this probability was <0.2 [22], and an “other spot” if neither threshold was met. In the subsequent phase, provinces assigned to each risk group in the first phase were further categorized into three trend patterns based on posterior estimates of exp. (*b*_*1i*_). An increasing trend relative to the overall trend was identified if *p* (*b*_*1i*_ > 0| hi, data) ≥ 0.8; a decreasing trend if *p* (*b*_*1i*_ > 0| hi, data) < 0.2; and a stable trend if 0.2 < *p* (b1*i* > 0|hi, data) < 0.8. Statistical analyses were performed using WinBUGS 1.4.3 (MRC Biostatistics Unit, University of Cambridge, UK).

#### 2.2.2. Age–Period–Cohort (APC) Analysis

The APC model is widely used in the fields of epidemiology research. This model facilitates the determination of net drift and local drift that represents overall time trends and specific time trends, while it also estimates the impact of the time dimensions: age, period, and birth cohort [23]. Within the APC framework, age and period intervals should maintain consistent temporal scales; consequently, utilization of 5‐year age groups requires corresponding 5‐year calendar periods. Hence, the timeframe spanning from 2005 to 2019 was separated into three 5‐year periods: 2005–2009, 2010–2014, and 2015–2019, and 16 age groups with a 5‐year interval, with the highest age group being ≥ 75 years. The analysis was performed using the APC model web tool (Biostatistics Branch, National Cancer Institute, Bethesda, MD. https://analysistools.nci.nih.gov/apc/).

#### 2.2.3. Optimal Parameters‐Based Geographical Detector (OPGD)

The OPGD model analyzes spatial variance to identify optimal parameter combinations for evaluating explanatory variables’ power. The Q‐statistic was employed to quantify the relative importance of explanatory variables through variance dispersion comparisons between the entire study area and variable‐defined strata [24]. The *Q* value of an influencing variable *v* is defined as:

Q=1−∑j=1MNv,jσv,j2Nvσv2,

where *Q* denotes the determining power of a factor, with values ranging from 0 to 1. It reflects the extent to which a risk variable or target factor contributes to heterogeneity. *j* represents the number of strata for an explanatory variable *v*, and M is the ideal strata level created by the discretization methods in the OPGD model. *N*_*v*_ and *σ* ^2^_*v*_ are the total unit numbers and variance of JE across the entire study area, and *N*_*v*_ and *σ* ^2^_*v*, *j*_ are the corresponding numbers and variances within the *j* ^th^ (*j* = 1,2 *… M*) substratum of variable *v*.

According to the OPGD model, a total of 16 indicators were selected as the explanatory variables of JE incidence in mainland China (Table 1). All variables were computed as annual averages and then were employed to evaluate their effects on JE incidence. The analysis was conducted using the “GD” package (version 10.8) in R software 4.4.3.

**Table 1:** Summary of meteorological factors, ecological factors, and socioeconomic factors of Japanese encephalitis in China, 2004–2020.

Driving factors	Variable	Average	5% percentile	50% percentile	95% percentile
Meteorological factors	Temperature (°C)	13.66	4.12	14.71	22.32
	Precipitation (mm)	8909.42	1662.91	7765.02	17584.05
	Relative humidity (%)	66.63	50.52	67.82	80.1
	Wind speed (m/s)	2.15	1.56	2.18	2.82

Ecological factors	NDVI	0.67	0.32	0.75	0.82
	Forest (%)	35.64	0.85	39.54	71.1
	Water (%)	2.55	0.30	1.4	11.17
	Cropland (%)	34.46	0.87	30.22	70.25
	Impervious (%)	6.48	0.01	2.59	27.37

Socioeconomic factors	Per capita GDP (RMB)	40176.46	9289.4	34,864	95,398
	RPT (10,000 persons)	72626.93	5808	57,637	199133.8
	Medical level (per 1000 population)	5.27	2.84	5.18	7.93
	Education level	8.72	6.85	8.77	10.68
	Built‐up area	1.82	0.02	0.73	8.51
	Population density (persons/sq.km)	436.23	7.93	270.59	1225.7
	Pig density (pigs/sq.km)	95.62	1.29	99.39	212.35

## 3. Results

### 3.1. Epidemiological Characteristics

From 2004 to 2020, a total of 43,857 cases of JE were reported in 31 provinces, China, with an annual incidence rate of 0.18 per 100,000 population and annual mortality rate of 0.01 per 100,000 population. The annual incidence rate per 100,000 population of JE decreased substantially, with a 95.24% reduction from 0.42 in 2004–0.02 in 2020 (Figure 1a). The distribution of JE exhibits marked seasonality, with a peak incidence from June to August. Prior to 2010, the low–latitude provinces reported higher incidence (peaking in Guizhou at 4.07 per 100,000 in 2006); thereafter, the high‐latitude provinces showed increasing incidence (peaking in Ningxia at 2.38 per 100,000 in 2018) (Figure 1b).

Figure 1The epidemiological features and demographic characteristics of Japanese encephalitis in China, 2004–2020. (a) The incidence of Japanese encephalitis in China (2004–2020). (b) Provincial mean annually incidence rate of Japanese encephalitis in China (2004–2020), sorted by descending province centroid latitude.

**Figure:**
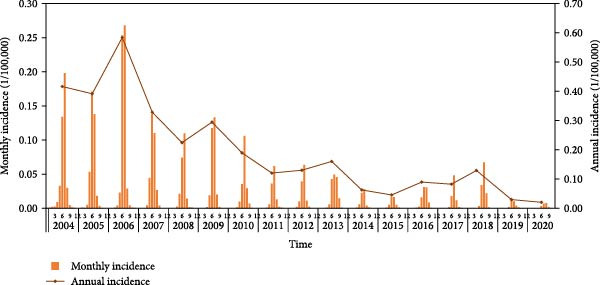
(a)

**Figure:**
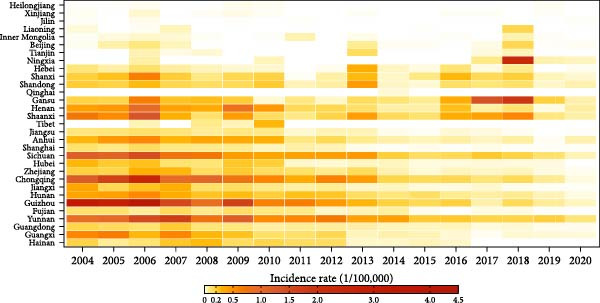
(b)

### 3.2. Spatial–Temporal Heterogeneity

**Figure 2:**
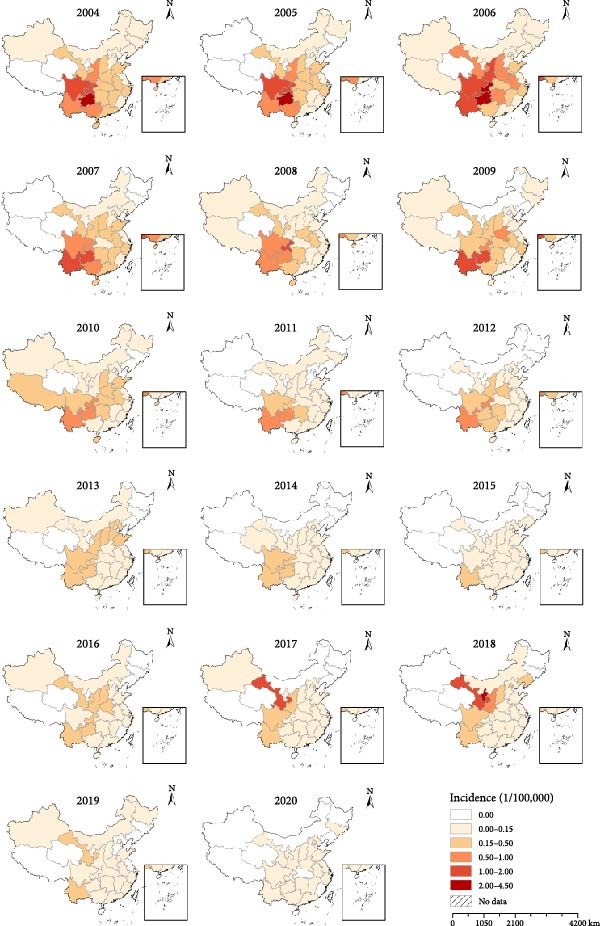
Spatial and temporal distributions of Japanese encephalitis at the provincial level in China, 2004–2020.

Between 2004 and 2020, the spatiotemporal distribution of JE in mainland China exhibited significant spatial and temporal heterogeneities (Figure 2). The higher JE incidence rates were observed in southwestern regions (Yunnan, Guangxi, Guizhou, Sichuan, and Chongqing), whereas the lower rates occurred in the northeastern region (Heilongjiang, Jilin, and Liaoning), as well as Tibet and Qinghai. Furthermore, JE transmission exhibited a distinctly northward expansion, with spread into northwestern regions. Notably, during 2017–2018, northwestern provinces (Shaanxi and Gansu) experienced outbreaks.

Figure 3a illustrated that 6 (19.35%), 5 (16.13%), and 20 provinces (64.52%) were identified as hot spots, cold spots, and other spots, respectively. The hot‐spot areas were mainly concentrated in southwestern China, while the cold‐spot areas were mainly located in northeastern China. Furthermore, the hot‐spot provinces such as Guangxi, Guizhou, Sichuan, Chongqing, and Hunan exhibited a downward trend (Figure 3b). Notably, Yunnan might be a potential high risk or hot‐spot even though it had a decreasing trend. Among the five cold spots (Jilin, Heilongjiang, Liaoning, Inner Mongolia, and Tianjin), all showed increasing trends. Meanwhile, among the other spots provinces, northern provinces (Beijing, Hebei, Shanxi, Shaanxi, Gansu, Qinghai, Ningxia, and Xinjiang) showed a rising trend in incidence. Nine southern provinces—Shanghai, Jiangsu, Zhejiang, Anhui, Fujian, Jiangxi, Henan, Hubei, and Hainan—had a declining trend. The remaining provinces, Guangdong and Tibet, followed the overall trend.

Figure 3Spatial–temporal heterogeneity of Japanese encephalitis in mainland China, 2004–2020. (a) The spatial relative risks of Japanese encephalitis at the provincial administrative units, (b) The distribution of hot and cold spots, and province‐level temporal trends of Japanese encephalitis.

**Figure:**
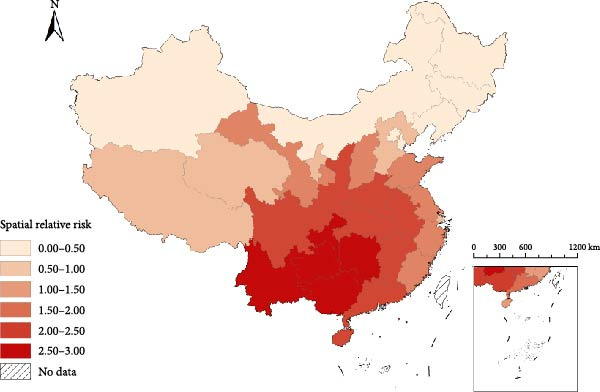
(a)

**Figure:**
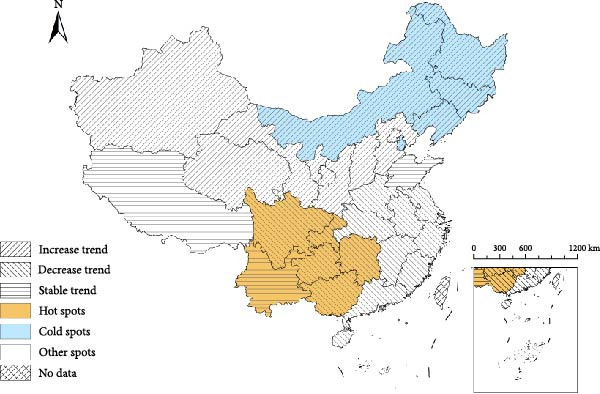
(b)

### 3.3. APC Model

The results of the APC model revealed the variations of JE incidence in China across different age groups and birth cohorts over the study period (Figure 4a,b). The incidence rate peaked in the 0–4 age group (4.33 per 100,000 population, 95% CI: 3.33–5.64) and then declined steeply through the 10–14 years old. Subsequently, the population aged ≥ 60 years, especially individuals aged 70–74 years (0.20 per 100,000 population, 95% CI: 0.13–0.28), exhibited a higher incidence, though still lower than the child population (0–14 years). From 2004 to 2010, children (0–14 years) consistently accounted for the majority of cases, peaking at 91% of total JE cases in 2005. Beginning in 2011, a sharp decline occurred, reaching a nadir of 21% in 2018. Conversely, case proportions among middle‐aged (15–59 years) and elderly (≥ 60 years) groups increased steadily from 2011 onward, collectively peaking at 79% of total cases in 2018 (Figure S2). The birth cohort (1956–1976) exhibited a relatively high risk of JE (Figure 4b). Birth cohort effects showed that the population born in 1966 had the highest risk of JE (RR = 1.13, 95% CI: 0.68–1.89), while the cohorts born before 1956 and after 1976 were significantly lower than the overall average risk of JE.

Figure 4Age and birth cohort effects on Japanese encephalitis incidence in China (2004–2020) by APC models. (a) The variations of age groups in the incidence of Japanese encephalitis. (b) The effects of the birth cohort on the relative risk of Japanese encephalitis.

**Figure:**
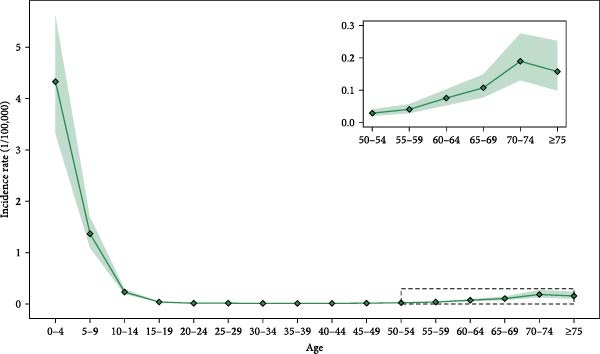
(a)

**Figure:**
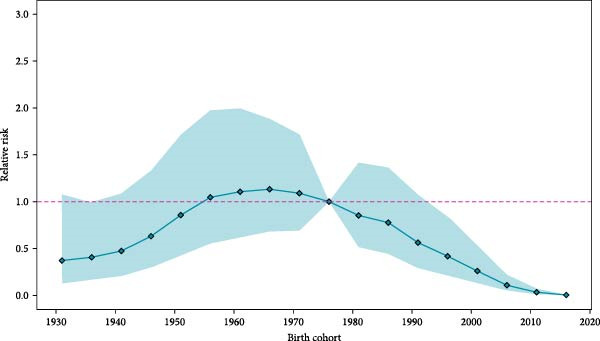
(b)

### 3.4. Risk Factors of JE

**Figure 5:**
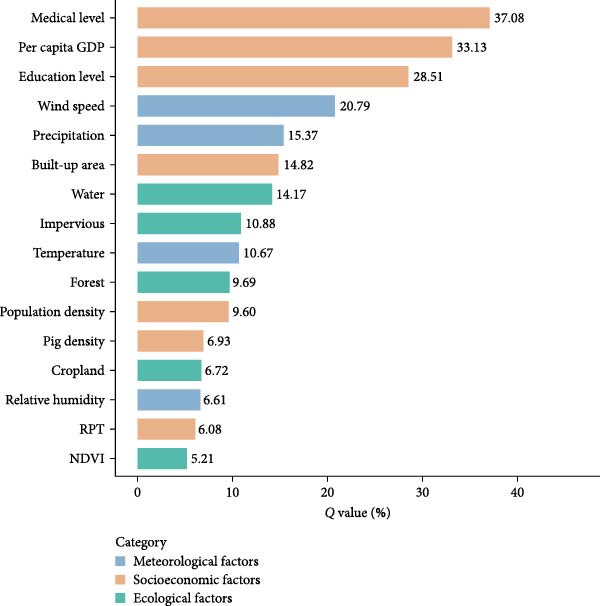
The Q value of driver factors in the spatial–temporal distribution of Japanese encephalitis in China, 2004–2020.

**Figure 6:**
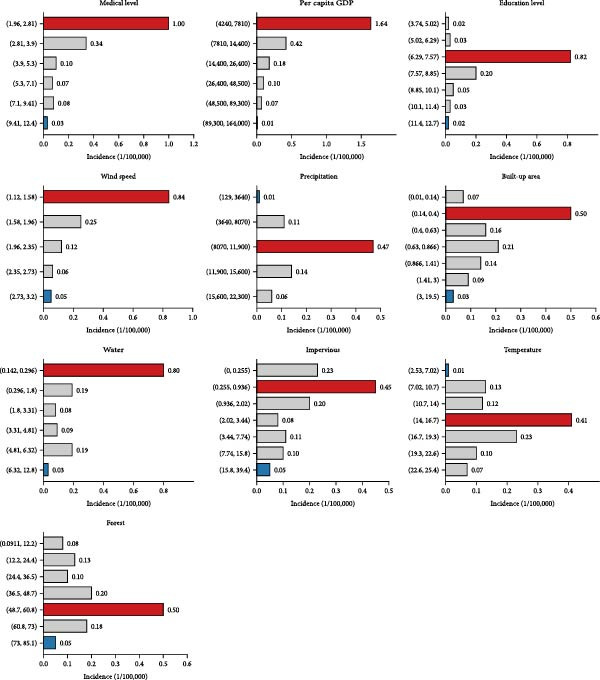
The different determinant effect of rank of the top 10 factors in the spatial–temporal distribution of Japanese encephalitis in China, 2004–2020.

The socioeconomic factors were the dominant factors of JE incidence in China (Figure 5). Medical level (*Q* value = 37.08%, *p*  < 0.01) exhibits the strongest association with JE incidence. The next dominant factor was the per capita GDP (*Q* value = 33.13%, *p*  < 0.01), followed by education level (*Q* value = 28.51%, *p*  < 0.01), wind speed (*Q* value = 20.79%, *p*  < 0.01), precipitation (*Q* value = 15.37%, *p*  < 0.01), built–up area (*Q* value = 14.82%, *p*  < 0.01), water (*Q* value = 14.17%, *p*  < 0.01), impervious (*Q* value = 10.88%, *p*  < 0.01), and temperature (*Q* value = 10.67%, *p*  < 0.01) (Table 2). In contrast, the other factors had relatively lower effects on the distribution of JE (*Q* values < 10%, respectively). Moreover, different levels of each environmental factor exerted heterogeneous effects on the incidence of JE (Figure 6).

**Table 2:** Q value and statistical significance of influencing factors for Japanese encephalitis.

Variable	Q value (%)	Sig. (*p*)
Medical level	37.08	<0.001
Per capita GDP	33.13	<0.001
Education level	28.51	<0.001
Wind speed	20.79	<0.001
Precipitation	15.37	<0.001
Built‐up area	14.82	<0.001
Water	14.17	<0.001
Impervious	10.88	<0.001
Temperature	10.67	<0.001
Forest	9.69	<0.001
Population density	9.60	<0.001
Pig density	6.93	<0.001
Cropland	6.72	<0.001
Relative humidity	6.61	<0.001
RPT	6.08	<0.001
NDVI	5.21	<0.001

## 4. Discussion

The study systematically examined the epidemiological trends and underlying drivers of JE across mainland China from 2004 to 2020. The principal findings are summarized as follows: The distribution of JE exhibited substantial spatial heterogeneity, and high‐risk clusters remained predominantly concentrated in southwestern China, with a noticeable northward expansion of Gansu and Shaanxi provinces. The highest disease burden occurred in children aged 0–14 years, followed by older adults aged ≥ 60 years. Moreover, socioeconomic factors were found to be inversely associated with JE risk distribution.

Based on the spatiotemporal distribution analysis of JE incidence from 2004 to 2020, JE outbreaks can be primarily divided into two phases. The first phase (2004–2010) witnessed a rapid decline in JE incidence, which is likely attributable to the implementation of vaccination policies [25]. A pivotal milestone occurred in 2008 when China incorporated the JE vaccine into the national immunization program, providing free vaccinations for children aged ≥ 8 years, excluding non‐endemic provinces such as Xinjiang, Qinghai, and Tibet [26]. The second phase (2011–2020) exhibited a fluctuating but overall declining trend, culminating in the lowest recorded incidence in 2020. However, there was a sudden increase in JE incidence in 2017 and 2018, particularly in the northwestern provinces of Gansu and Shaanxi. This unexpected increase is likely attributable to inadequate enforcement and/or insufficient vaccination coverage within specific subpopulations in these regions [8].

The study revealed significant spatial heterogeneities of JE risk across China. Incidence rates were highest in the mid‐latitude provinces, while provinces at both high and low latitudes exhibited substantially lower disease burdens. The distribution patterns of JE in China demonstrate the significant influence of climate change and human activities that have exacerbated the transmission risk of mosquito‐borne diseases [14]. Specifically, increased human mobility and global warming have jointly facilitated the dispersal and range expansion of *Culex* mosquito vectors across China. Consequently, these vectors are now distributed throughout all provinces of China, with the exception of Xinjiang, Tibet, and Qinghai [27]. This widespread establishment indicates that mid‐latitude regions are increasingly emerging as critical areas for JE prevention and control efforts.

Results from the Bayesian spatiotemporal model reveal divergent temporal trends: JE incidence is increasing across northern provinces (excluding Tibet), while southern provinces generally show declining trends (with the notable exception of Yunnan). Tibet’s unique geographical conditions (including high altitude and low temperatures) are not conducive to JEV transmission. Meanwhile, Yunnan persists as the sole hot–spot province maintaining stable high incidence, necessitating sustained prioritization for prevention and control due to its economic constraints and agricultural practices. Given Yunnan’s proximity to JE‐endemic countries (e.g., Myanmar and Laos), enhanced surveillance of imported cases at border crossings is crucial to mitigate cross‐border transmission risks [28, 29]. The rising incidence in northern provinces correlates with slower economic development, expanded pig production, and suboptimal implementation of vaccination policies [30].

The APC model analysis revealed significantly elevated JE risk in the 0–14 age group, consistent with previous studies identifying children as a susceptible population [9]. Notably, individuals aged ≥ 60 years also demonstrated elevated JE incidence risk, potentially attributable to diminished immunity and increased JEV susceptibility in the elderly population [31]. Furthermore, the elevated risk observed among children and the elderly may be attributed to rural‐to‐urban migration of working‐age adults, resulting in residual concentrations of these vulnerable populations in rural areas. This demographic shift leads to heightened exposure to mosquito vectors, subsequently increasing infection rates in both groups. This pattern aligns with the documented outbreaks among unvaccinated adults, exemplified by the 2017–2018 epidemic in Gansu Province [32]. Therefore, the provinces should implement tailored vaccination programs aligned with local epidemiology, prioritize rural policy enforcement, and enhance surveillance among adults and the elderly. Expanding routine immunization to include middle‐aged and elderly populations should be considered to strengthen population–wide protection [6].

As a mosquito‐borne infectious disease, JE transmission is generally considered primarily influenced by meteorological factors. However, our study revealed that in China, socioeconomic factors exhibit a significant negative association with JE incidence at the province level. This finding demonstrates that within China’s unique environmental context, socioeconomic development outweighs meteorological influences as the dominant force shaping JE transmission dynamics.

Socioeconomic factors such as medical level and educational level were the dominant factors on JE incidence, indicating that medical testing capabilities, disease prevention education, and vaccination are key mechanisms suppressing transmission. Moreover, the research indicates that southeastern China, once high‐incidence areas for JE, has experienced a notable decline in incidence following the implementation of effective vaccination programs, and their current incidence rates are well‐controlled [33]. Evidence indicates wind speed negatively affects mosquito‐borne disease incidence [34], which is similar to our finding of a negative association between wind speed and JE incidence. We found a nonlinear association between temperature and JE incidence, which was in line with the previous study [35]. Among ecological factors, water bodies exhibited a negative association with JE incidence, likely reflecting the breeding site preferences of the principal JE vector, *Culex* mosquitoes, which preferentially breed in small aquatic habitats.

Strengthening veterinary surveillance, promoting vaccination, and reducing high‐risk human–mosquito contact are essential to effectively reduce the JE disease burden [36]. Moreover, anticipating and early detecting of emerging JE cases are key to controlling its spread. Given the lack of treatment for JE, vaccination remains the most effective strategy to combat JEV transmission and reduce human exposure [8]. In this context, China’s JE vaccination pattern aligns with early‐adopting countries like Japan and South Korea [37, 38], where sustained high childhood immunization has shifted the disease burden predominantly to adults and the elderly. This transition highlights the need for booster vaccination strategies targeting older adults population. Given the socioeconomic and environmental disparities between northern and southern China, and considering the fragmented coverage observed in some Southeast Asian countries due to urban–rural divides [39, 40], it is crucial to maintain robust vaccination programs while strengthening northern health systems. Specifically, immunization policies should be consistently applied in established southern high‐risk provinces (Sichuan, Guizhou, Yunnan, Chongqing) while being enhanced in emerging northern risk areas (Gansu, Shaanxi). Additionally, refined age‐specific vaccination strategies are urgently needed for the elderly population facing growing JE infection risks. As JE is a mosquito‐borne disease, the application of mosquito larvicides and larval habitat elimination is therefore critical to counter vertical transmission [41].

Several limitations inherent in the study should be acknowledged. First, underreporting may exist within the disease surveillance system, particularly prior to the widespread adoption of digital reporting platforms. Hence, undernotification remains a persistent concern that could affect data completeness. Additionally, due to the absence of available data in public health science databases, we were unable to collect detailed demographic information such as gender, occupation, and specific residential locations, which restricted further exploration of more detailed epidemiological characteristics of JE. Furthermore, key determinants like vaccination coverage were omitted due to data constraints. This absence may have inflated the estimated influence of some socioeconomic and meteorological drivers. Therefore, future research should prioritize collaborative data collection and the implementation of refined analytical methodologies to address this critical evidence gap.

## 5. Conclusion

Our findings reveal significant spatiotemporal heterogeneity in the distribution of JE across mainland China, primarily driven by meteorological and socioeconomic factors. Thus, JE control and prevention measures should be strengthened by integrating spatiotemporal targeting and the aforementioned influencing factors to formulate targeted public health interventions.

## Ethics Statement

Ethical approval was not needed for the study as it is based on routine surveillance.

## Disclosure

The funders had no role in study design, data collection and analysis, decision to publish, or preparation of the manuscript.

## Conflicts of Interest

The authors declare no conflicts of interest.

## Author Contributions

**Junze Du and Rui Li:** conceptualization, methodology, writing – original draft. **Peng Li and Ting Fu:** methodology, data curation, writing – review and editing. **Zurong Yang and Bo Wen:** methodology. **Zhijia Sun and Guzhengyue Zheng:** writing – review and editing. **Haiyan Zhou and Hongxia Tan:** software, data curation, visualization. **Kun Liu:** conceptualization, writing – review and editing, project administration, supervision. Junze Du and Rui Li have contributed equally to this work.

## Funding

This study was supported by the National Natural Science Foundation of China (Grant 82473689) and the National Natural Science Foundation of Shaanxi Province (Grant 2025JC–YBQN–1103).

## Supporting Information

Additional supporting information can be found online in the Supporting Information section.

## Supporting information


**Supporting Information** Table S1: Classification of Chinese provinces by region and latitudinal zones. Table S2: The description of influencing factors. Figure S1: The relative risk of Japanese Encephalitis in China, annually from 2004 to 2020. Figure S2: The proportion of Japanese Encephalitis by age group in China, annually from 2004 to 2020.

## Data Availability

The data that support the findings of this study are available from the corresponding author upon reasonable request.
