# Supplementary material for: Characterizing Epidemiological Trends and Associated Factors of Japanese Encephalitis in China: Insights From a 17–Year National Surveillance Analysis
Source: Transbound Emerg Dis. 2025 Dec 28;2025:8854015. doi: 10.1155/tbed/8854015 (PMC12745838; doi:10.1155/tbed/8854015)
Supplement: Supplementary file 1 — Supporting Information Table S1: Classification of Chinese provinces by region and latitudinal zones. Table S2: The description of influencing factors. Figure S1: The relative risk of Japanese Encephalitis in China, annually from 2004 to 2020. Figure S2: The proportion of Japanese Encephalitis by age group in China, annually from 2004 to 2020. [file TBED-2025-8854015-s001.docx]

Appendix

1. Zones

In this study, the 31 provinces of mainland China were grouped into the northern provinces and southern provinces based on a prior study [1] and the Qinling–Huaihe River line that serves as a climatic demarcation in China. Additionally, we reclassified these provinces into three latitude zones according to the capital’s location of each province and the areas of each province, combined with general cognition of the natural environment.

**TABLE S1: Classification of Chinese provinces by region and latitudinal zones.**

| **Zone** | **Count(proportion)** | **Provinces** |
| --- | --- | --- |
| Northern China | 16 (51.61%) | Heilongjiang, Jilin, Xinjiang, Liaoning, Inner Mongolia, Beijing, Tianjin, Ningxia, Hebei, Shanxi, Shandong, Qinghai, Gansu, Henan, Shaanxi, Tibet |
| Southern China | 15 (48.39%) | Jiangsu, Anhui, Shanghai, Sichuan, Hubei, Zhejiang, Chongqing, Jiangxi, Hunan, Guizhou, Fujian, Yunnan, Guangdong, Guangxi, Hainan |
| High latitude (> 40°N) | 5 (16.19%) | Heilongjiang, Jilin, Xinjiang, Liaoning, Inner Mongolia |
| Mid latitude (30°N~40°N) | 16 (51.61%) | Beijing, Tianjin, Ningxia, Hebei, Shanxi, Shandong, Qinghai, Gansu, Henan, Shaanxi, Jiangsu, Anhui, Shanghai, Sichuan, Hubei, Zhejiang |
| Low latitude (< 30°N) | 10 (32.26%) | Tibet, Chongqing, Jiangxi, Hunan, Guizhou, Fujian, Yunnan, Guangdong, Guangxi, Hainan |

1. Description of influencing factors.

**TABLE S2: The description of influencing factors.**

| Variable | Description |
| --- | --- |
| Temperature | Mean annual temperature |
| Precipitation | Mean annual precipitation |
| Relative humidity | Average years of schooling |
| Wind speed | Mean annual wind speed |
| NDVI | Normalized Difference Vegetation Index |
| Forest | Forest area |
| Water | Water area |
| Cropland | Cropland area |
| Impervious | Impervious surface area |
| Per capita GDP | Per capita Gross Domestic Product |
| RPT | Passenger Traffic by Region |
| Medical level | Medical technical personnel |
| Education level | Cropland area |
| Built–up area | Urban built-up area |
| Population density | The number of persons per unit area |
| Pig density | The number of pigs per unit area |

*Education level: Average years of schooling was computed as:

*E_i_*​: Population count within educational attainment category *i*.

*Y_i_​*: Years of schooling assigned to category *i.*

*P_aged_* ≥6​: Total population aged ≥6 years.

*Y_i_* denotes years assigned per education level:

Illiterate=0, Primary=6, Junior secondary=9, Senior secondary (including vocational) =12, Higher secondary (including Normal and Short–cycle Courses)=16.

1. Additional figures


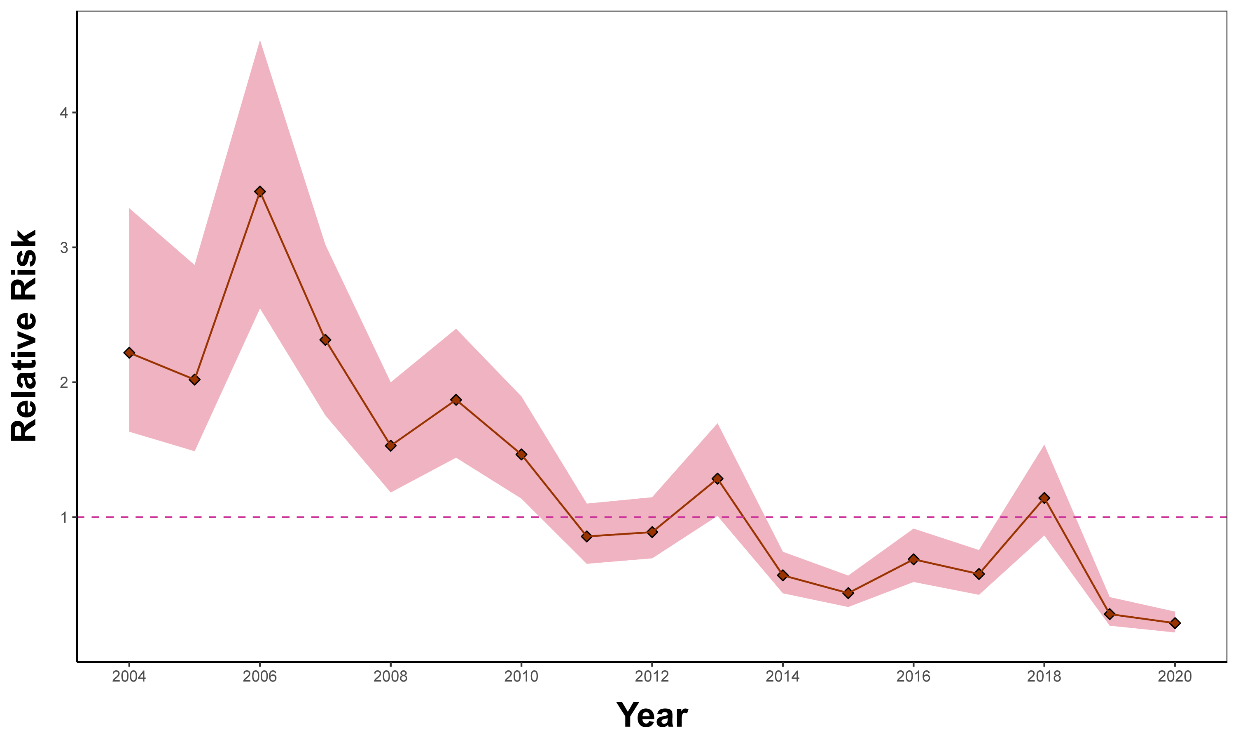


**FIGURE S1: The relative risk of Japanese Encephalitis in China, annually from 2004 to 2020.**


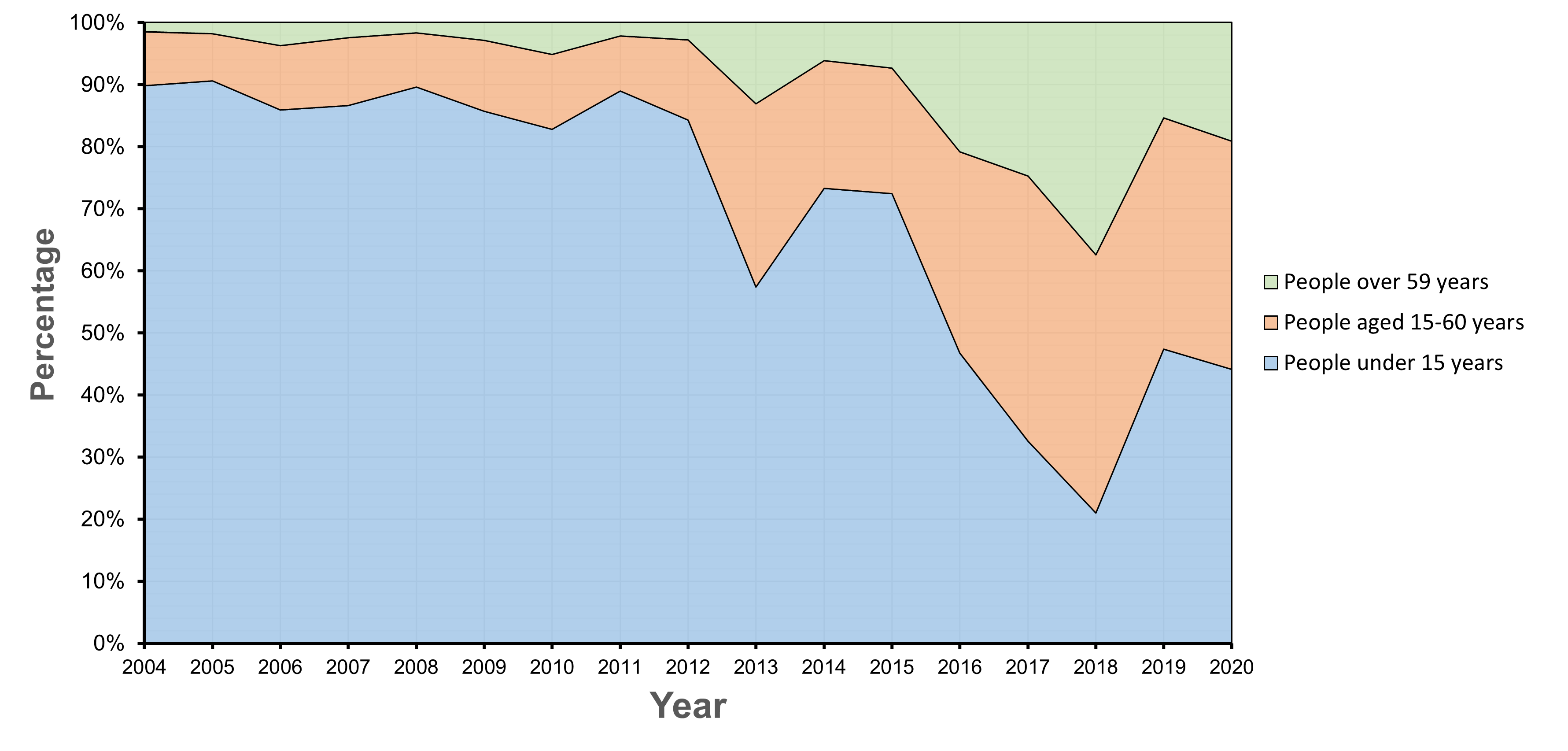


**FIGURE S2: The proportion of Japanese Encephalitis by age group in China, annually from 2004 to 2020.**

**Reference**

[1] F. Liu, Y. Yi, Y. Song, et al. “Epidemiology of hand, foot, and mouth disease outbreaks before and during availability of EV-A71 vaccine in China's mainland: analysis of outbreak surveillance data from 2011 to 2023,” *Lancet Reg Health West Pac* 59, no. (2025): 101603.
